# Effects of Peptide Thanatin on the Growth and Transcriptome of *Penicillium digitatum*

**DOI:** 10.3389/fmicb.2020.606482

**Published:** 2020-12-14

**Authors:** Guirong Feng, Xindan Li, Wenjun Wang, Lili Deng, Kaifang Zeng

**Affiliations:** ^1^College of Food Science, Southwest University, Chongqing, China; ^2^Research Center of Food Storage and Logistics, Southwest University, Chongqing, China

**Keywords:** peptide thanatin, citrus, *Penicillium digitatum*, transcriptome, antifungal

## Abstract

*Penicillium digitatum* is the most damaging pathogen provoking green mold in citrus fruit during storage, and there is an urgent need for novel antifungal agents with high efficiency. The aim of this study was to investigate the antifungal effects of peptide thanatin against *P. digitatum* and the molecular mechanisms. Results showed that peptide thanatin had a prominent inhibitory effect on *P. digitatum* by *in vitro* and *in vivo* test. A total of 938 genes, including 556 downregulated and 382 upregulated genes, were differentially expressed, as revealed by RNA-seq of whole *P. digitatum* genomes analysis with or without thanatin treatment. The downregulated genes mainly encoded RNA polymerase, ribosome biogenesis, amino acid metabolism, and major facilitator superfamily. The genes associated with heat shock proteins and antioxidative systems were widely expressed in thanatin-treated group. DNA, RNA, and the protein content of *P. digitatum* were significantly decreased after thanatin treatment. In conclusion, thanatin could inhibit the growth of *P. digitatum*, and the underlying mechanism might be the genetic information processing and stress response were affected. The research will provide more precise and directional clues to explore the inhibitory mechanism of thanatin on growth of *P. digitatum*.

## Introduction

The large losses of fruit products during the postharvest period are usually caused by pathogenic plant diseases, and the blue or green molds caused by *Penicillium* species are particularly serious (Palou, 2018; Pétriacq et al., 2018). *Penicillium digitatum* is one of the most destructive pathogens, leading to about 90% of total crop losses, and is the causal agent of green mold disease in citrus fruit (Zhu et al., 2017). Currently, the use of chemical fungicides is the main way to control this pathogen. However, widespread overuse of chemical antibiotics increases the potential risks of new antibiotic-resistant strains, and antibiotic pollution may also harm environment and human health (Sanchez-Torres and Tuset, 2011; Erasmus et al., 2015). There is therefore an urgent need to explore a new and effective method to control citrus green mold disease caused by *P. digitatum*.

 Recently, the application of novel biological methods has attracted a lot of attentions (Tao et al., 2014; Osman et al., 2016; Perez et al., 2016). Notably, antimicrobial peptides (AMPs) are considered as potential alternates to traditional fungicides due to their lower drug-resistance risks and less toxic to humans and environment. AMPs have a broad-spectrum antimicrobial ability against fungi and bacteria. They are characterized as relative short amino acid sequence residues, cationic charge (often) or anionic charge (rare), and structural diversity (Kang et al., 2017; Koehbach and Craik, 2019). Thanatin (GSKKPVPIIYCNRRTGKCQRM) is a kind of cationic AMP isolated from the insect *Podisus maculiventris* with the structure of β-hairpin induced by the residue of disulfide bridge of two cysteine residues. It has the prominent broad-spectrum antimicrobial activity, low hemolyticity, and cytotoxicity (Fehlbaum et al., 1996; Edwards et al., 2016), making it a potential candidate for the alternatives of chemical fungicides.

Many attempts have been made to uncover the perplexing microbiocidal mechanisms of AMPs. The most classical one is the membrane-lytic mechanism: AMPs would form pores on membranes or cause membrane disruption and further lead to the loss of integrity of cell barrier, which may promote the cytoplasmic leakage or even cell death (Lee et al., 2016; Shagaghi et al., 2018). Most cationic peptides can target with negatively charged cell membrane due to their amphipathic and cationic nature by the electrostatic interactions (Lei et al., 2019). While the binding of some anionic AMPs to target cells mainly depends on their amphipathic properties, which help them overcome the repulsive forces between these AMPs and negatively charged cell membrane (Cytryńska and Zdybicka-Barabas, 2015). Moreover, AMPs may directly act on intracellular target or induce various cell metabolic disorders (Le et al., 2017). For example, some peptides could inhibit DNA, RNA, and protein synthesis and thus block their growth (Patrzykat et al., 2002; Mardirossian et al., 2014; Braffman et al., 2019). Likewise, Indolicidin (IR13) could inhibit the replication and transcription process by interacting with DNA (Ghosh et al., 2014).

For thanatin, the possible antimicrobial mechanisms have been proposed by several reports. Thanatin can interact with the outer membrane lipopolysaccharide (LPS) and further cause cell agglutination (Sinha et al., 2017). The dual action mechanisms on NDM-1-producing bacteria occurs in the disruption of the outer membrane and the inhibition of NDM-1 enzyme activity (Ma et al., 2019). It has also been reported that thanatin is strong in binding to the DNA of *Geotrichum citri-aurantii*, but weak in destroying the cell membrane (Liu et al., 2019). However, there is still lack of evidence on the antimicrobial mechanisms of thanatin at the molecular level.

RNA-seq, with an advantage of high sequencing accuracy and depth, can analysis the whole genome scale of samples to reveal changes in cellular processes or metabolic pathways when exposed to variable environmental conditions, which has been successfully used in the gene transcriptional analysis of plant pathogens under fungicide stress (OuYang et al., 2016; Zhou et al., 2018b; Wang et al., 2019). The aim of our work was to investigate the molecular action of thanatin on *P. digitatum* using high-throughput RNA-seq technique.

## Materials and Methods

### Fungal Cultivation and Peptide Synthesis

The fresh spores of *P. digitatum* were scraped from potato dextrose agar plates (PDA: 200 g L^–1^ potato, 20 g L^–1^ glucose, 20 g L^–1^ agar powder) after cultured 7 day at 25°C. The spore suspension was filtered through a gauze, washed with sterile water, and counted with a hemocytometer. The mycelia were obtained by adding 1 mL fungal spore suspension (1 × 10^5^ CFU mL^–1^) to 100 mL potato dextrose broth (PDB: 200 g L^–1^ potato, 20 g L^–1^ glucose), shaking it (160 rpm), and culturing it at 25°C for 48 h.

Thanatin (GSKKPVPIIYCNRRTGKCQRM) with >95% purity was synthesized by GenScript Corporation (Nanjing, China) through the solid-phase method using N-(9-fluorenyl) methoxycarbonyl (Fmoc) chemistry. The peptide was provided as white lyophilized (freeze-dried) powder in multiple vials. The stock solution (1 m mol L^–1^) was prepared in ultrapure water and adjusted to the suitable concentration before the test and stored at −20°C.

### Antifungal Effects on Spore Germination and Survival

The germination inhibition effect of thanatin on spores was determined by a dose–response curve using a 96-well assay plate as previously described (Wang et al., 2018). In each well, the spore suspensions that were 20-fold-diluted in PDB (180 μL, 1 × 10^4^ CFU mL^–1^) were mixed with 20 μL thanatin solution at the final concentrations of 0, 0.5, 1, 2, 4, 8, 16, or 32 μmol L^–1^, respectively, and the mixture was incubated at 25°C for 48 h. Absorbance was measured at OD_600_ using the Multiskan Spectrum microplate spectrophotometer (BioTek Instruments, Inc., United States). The minimum inhibitory concentration (MIC) value was defined as the lowest concentration of peptide at which no visible growth of spore can be observed in well after 48 h.

The inhibition effect of conidia survival was conducted as previously described with a few modifications (Liu et al., 2019). The spore suspensions (2 × 10^3^ CFU mL^–1^) were exposed to the peptide at final concentration of 0, 2, 4, 8, 32, 64, and 128 μmol L^–1^ for 16 h in a 2 mL sterile microcentrifuge tube. The treatment solution 50 μL was evenly spread on PDA solid medium. After incubation at 25°C for 36–48 h, the spore survival rate (%) was calculated by counting conidia of each plate using the following formula.

$$\text{Spore}\text{survival}\text{rate}\left(\%\right)=\text{Spore}\text{number}\left(\frac{\text{treatment}}{\text{control}}\right)\times 100\%$$

The time-kill kinetic assays were carried out using the above-mentioned counting method. The tubes containing spore suspension (2 × 10^3^ CFU mL^–1^) and thanatin of 2, 4, 8, 32, 64, and 128 μmol L^–1^ were incubated for 15, 30, 60, 120, 240, 360, and 540 min; the control group (shown as 0 min) was treated with sterile water and then spread on PDA plates and incubated at 25°C for 36–48 h to calculate the survival rate. All the determinations were performed in triplicate. The minimum fungicidal concentration (MFC) value was defined as the lowest concentration of peptide at which the spore survival rate <1%.

### Fruit Decay Test

The fruit decay test was carried out on olinda valencia oranges (*Citrus sinensis* (L.) Osbeck) as previously described with a few modifications (Wang et al., 2018). Fruit was harvested at a local orchard (Zhongxian, Chongqing), and was selected for similar size, color, maturity, and free mechanical injury. The oranges were softly scrubbed with gauze and sterilized by 2% (v/v) sodium hypochlorite for 2 min, washed twice with water, and then dried in air at room temperature. The fruit surface was further sterilized with 75% alcohol and wounded with two wounds (4 mm deep and 3 mm wide) located at the fruit equator using a sterile nail. The *P. digitatum* spore suspensions (5 × 10^4^ CFU mL^–1^) were treated with thanatin at 0 (sterile water), 8 μmol L^–1^, and 128 μmol L^–1^ for 2 h at room temperature. Afterward, 10 μL mixture was inoculated into each fruit wound. When the mixture was absorbed, the fruit was individually packed with plastic bags and then stored at 25°C and at 90–95% relative humidity. The disease incidence and lesion diameter were recorded after 3–6 days of inoculation. There were 15 fruit in each treatment, and the experiment was conducted with three replicates.

### Treatment and RNA Extraction

The mycelia cultured in PDB at 25°C for 2 day were washed twice with phosphate buffer saline (PBS, pH 7.0) on a sterile funnel. In order to prevent RNA degradation and to establish a cDNA library, the mycelia were rapidly frozen in liquid nitrogen immediately after the treatment with PBS (as control) or with thanatin (4 μmol L^–1^) in PBS for 2 h. Total RNA extraction and RNA-seq analysis were carried out by Beijing Genomics Institute (BGI) Co., Ltd. The total RNA was extracted by TRIzol regent (Invitrogen, United States) according to the manufacturer’s instruction. The purity was quantified by a NanoPhotometer spectrophotometer (IMPLEN, CA, United States). The degradation was checked using 1% agarose gel electrophoresis. The RNA integrity number (RIN) and concentration were measured by Agilent 2100 Bioanalyzer (Agilent Technologies, CA, United States). Three biological replicates for each group were performed for RNA extraction and RNA-seq analysis.

### The cDNA Library Construction

The mRNA was enriched from total RNA by using the oligo (dT) magnetic beads and being exposed to divalent cations and then randomly divided into small fractions. The fragmented mRNAs were collected and carried out following the manufacturer’s instructions of NEBNext^®^ Ultra^TM^ RNA Library Prep Kit for Illumina^®^. Briefly, the first-strand cDNA was synthesized from mRNA template, with random oligonucleotide primers and M-MuLV reverse transcriptase. Next, dNTPs were used as primers for the second-strand cDNA in the presence of DNA polymerase I. The double stranded cDNA was then subjected to end repair, poly A-addition, adaptor ligation, and screened for cDNA with a length of 250–300 bp. Initially, the library was quantified by Qubit2.0 Fluorometer. Afterward, the cDNA library was constructed using cDNA purified with AMPure XP beads, which was amplified by PCR. The insert size and effective concentration of the final library were evaluated by Agilent 2100 bioanalyzer and qualified by **Q**uantitative RT PCR (qRT-PCR).

### Bioinformatics Analysis

Quality control for the raw sequencing data was checked by filtering low quality reads, checking for sequencing error, and censoring rate of GC content distribution. The clean reads were mapped to reference genomes using hisat2 software (version 2.0.5), and annotated with *P. digitatum Pd1* (CECT: 20795, GCA 000315645)^1^. The expression levels were normalized as Fragments Per Kilobase of exon model per Million mapped reads (FPKM) by featureCounts software (version 1.5.0-p3). The correlation coefficient of all genes among the three biological replicates in each group was performed with Pearson method on FPKM value. For selection of differentially expressed genes (DEGs), a normalization step was firstly performed by the DESeq2 software (version 1.16.1) with the read count as the inputting data. The *p*-value was based on negative binomial distribution model, and the false discovery rate (FDR, the frequent form was padj) was calculated by BH method, which was used to select the DEGs. Finally, the following criteria of DEGs were used: |log2(Fold Change)| > 0 and padj < 0.05. (padj value was the multiple hypothesis-corrected *p* value). The clusterProfiler software (version, 3.4.4) was applied for the functional enrichment analysis of DEGs mapped into the database of GO (Gene Ontology) and KEGG (Kyoto Encyclopedia of Genes and Genomes).

### Real-Time Fluorescence Quantitative qRT-PCR Validation

The mycelia of *P. digitatum* treated at 0 or 4 μmol L^–1^ were used for extracting total RNA for qRT-PCR analysis using the above-mentioned method, and qualified by Nanodrop 2000 Spectrophotometer (Thermo-Fisher scientific Inc.,Wilmington, DE, United States). The cDNA was reverse transcribed from total RNA at the same insert amount (600 ng) using a kit of PrimeScript^TM^ RT reagent Kit (RR047Q, Takara, Japan). The reaction mixture was prepared in a 384-well plate containing 1 μL of cDNA template, 1 μL primer sets (10 μmol L^–1^), 10 μL of iTaq^TM^ Universal SYBR^®^ Green Supermix (Cat. 1725121, BIO-RAD, United States) and 7 μL double distilled water. The thermal cycling conditions were as follows: 95°C for 60 s, followed by 40 cycles of 95°C for 5 s and then 60°C for 30 s. The melt curve was performed in a 65–95°C range with increments of 0.5°C every 5 s. The relative expression level was validated on CFX Real-Time PCR Detection Systems (CFX96, BIO-RAD, United States), analyzed by the 2^–ΔΔ*Ct*^ method. Briefly, the Δ Ct value of target gene was normalized by the Ct mean value of the endogenous control, the ΔΔ Ct was calculated, respectively, by the subtraction from the Δ Ct between the control and treated samples. The experiments were performed in triplicate, and results were represented as mean values of 2^–ΔΔ*Ct*^ ± SD. The primer pair of actin gene was obtained (OuYang et al., 2016), and other specific primer pairs were designed in Primer-Blast^2^; these are listed in Supplementary Table 1.

### Determination of DNA and RNA Contents

The DNA and RNA contents were quantified by 4′,6-diamidino-2-phenylindole (DAPI) binding method according to the methodology Wang et al., 2010), with some modification. DAPI is a fluorescent dye that could emit a bule fluorescence by effectively penetrating to the cells and binding to the minor groove of double–stranded DNA and the AU base pairs of RNA. The fresh spore suspensions (5 × 10^4^ CFU mL^–1^, 5% PDB) were incubated for 12 h at 25°C and then treated with thanatin (0, 0.5, 1, 2, 4, 8, and 32 μmol L^–1^) at 25°C for 2 h and 6 h, respectively. The 50 μL reaction mixture with an equal volume of DAPI (C1005, Beyotime, China) was added into a well of fluorescence plate and incubated for 10 min in dark. The fluorescence of binding of DAPI to DNA and RNA in cells was monitored using the Multiskan Spectrum microplate spectrophotometer (BioTek Instruments, Inc., United States) with the following program: excitation wavelength of 364 nm and 400 nm, respectively, and emission at 460 nm. The experiment was repeated three times.

### Determination of Soluble Protein Contents

The test was conducted with some modifications (Chen et al., 2019). The *P. digitatum* spore suspensions (1 × 10^5^ CFU mL^–1^, 5% PDB) were cultured for 12 h at 25°C, and then incubated with tahantin treatment (0, 1, 2, 4, and 8 μmol L^–1^) at 25°C for 2 h and 6 h, respectively. Cell lysis was performed by adding 0.4 *g* glass beads in each mixture (600 μL). The sample was then placed on ice and vortexted for 30 s periodically for 15 min, until the mixture was clear. After centrifugation at 10,000 × *g* for 20 min at 4°C, the 100 μL supernatant was stained with an equal volume of Coomassie Brilliant Blue (G-250, C8420, Solaribo, China) for 5 min. The mixture was measured at 595 nm using the Multiskan Spectrum microplate spectrophotometer (BioTek Instruments, Inc., United States). The soluble protein contents were determined via a Bradford assay using BSA as the standard. The experiment was repeated three times.

### Statistical Analysis

Apart from the experiments analyzed by the bioinformatics software, other data were expressed as mean ± SD by measuring three independent replicates using SPSS 20 software (SPSS Inc., United States). The differences of the data were compared with the variance (ANOVA), using Duncan’s multiple range test at *p* < 0.05. The figures were produced using GraphPad Prism 8.0 (GraphPad Software Inc., San Diego, CA, United States).

## Results

### Antifungal Effects of Thanatin on *P. digitatum in vitro*

**FIGURE 1:**
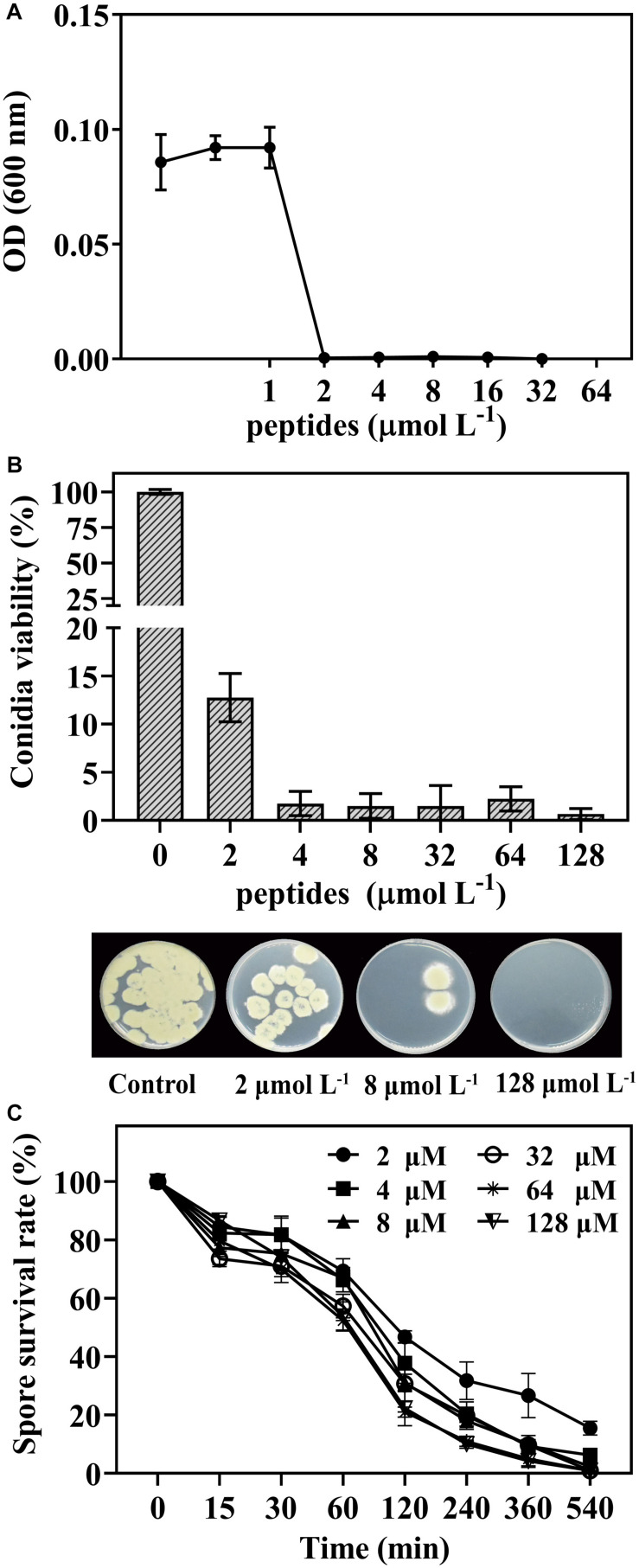
The dose-response curves of *P. digitatum* when exposed to thanatin for 48 h **(A)**. Conidia viability of *P. digitatum* exposed to thanatin at 0, 2, 4, 8, 32, 64, and 128 μmol L^–1^ after 16 h of incubation, and the image was the *P. digitatum* spore incubated for 3 days **(B)**. Survival rate of *P. digitatum* spores after treated with thanatin for 15, 30, 60, 120, 240, 360, and 540 min at different concentrations, the control group (shown as 0 min) was treated with sterile water **(C)**. The results were shown as the mean ± SD (*n* = 3).

The germination and survival of *P. digitatum* spores as affected by peptide thanatin were shown in Figure 1. Thanatin effectively inhibited the conidia germination with the MIC of 2 μmol L^–1^ (Figure 1A). Thanatin also showed high fungicidal activity after treatment for 16 h, the survival rate of conidia decreased to 12% at 2 μmol L^–1^ treatment, and the MFC was 128 μmol L^–1^ with the conidia survival rate < 1% (Figure 1B). Furthermore, higher treatment concentration or longer time resulted in higher lethality on *P. digitatum* (Figure 1C). The spore survival rate was decreased to approximately 47% after being incubated with thanatin at 2 μmol L^–1^ for 2 h and further decreased to 38, 31, and 22% when at 4, 8, and 128 μmol L^–1^ for 2 h, respectively.

### Effect of Thanatin on *P. digitatum* Infections on Citrus Fruit

**FIGURE 2:**
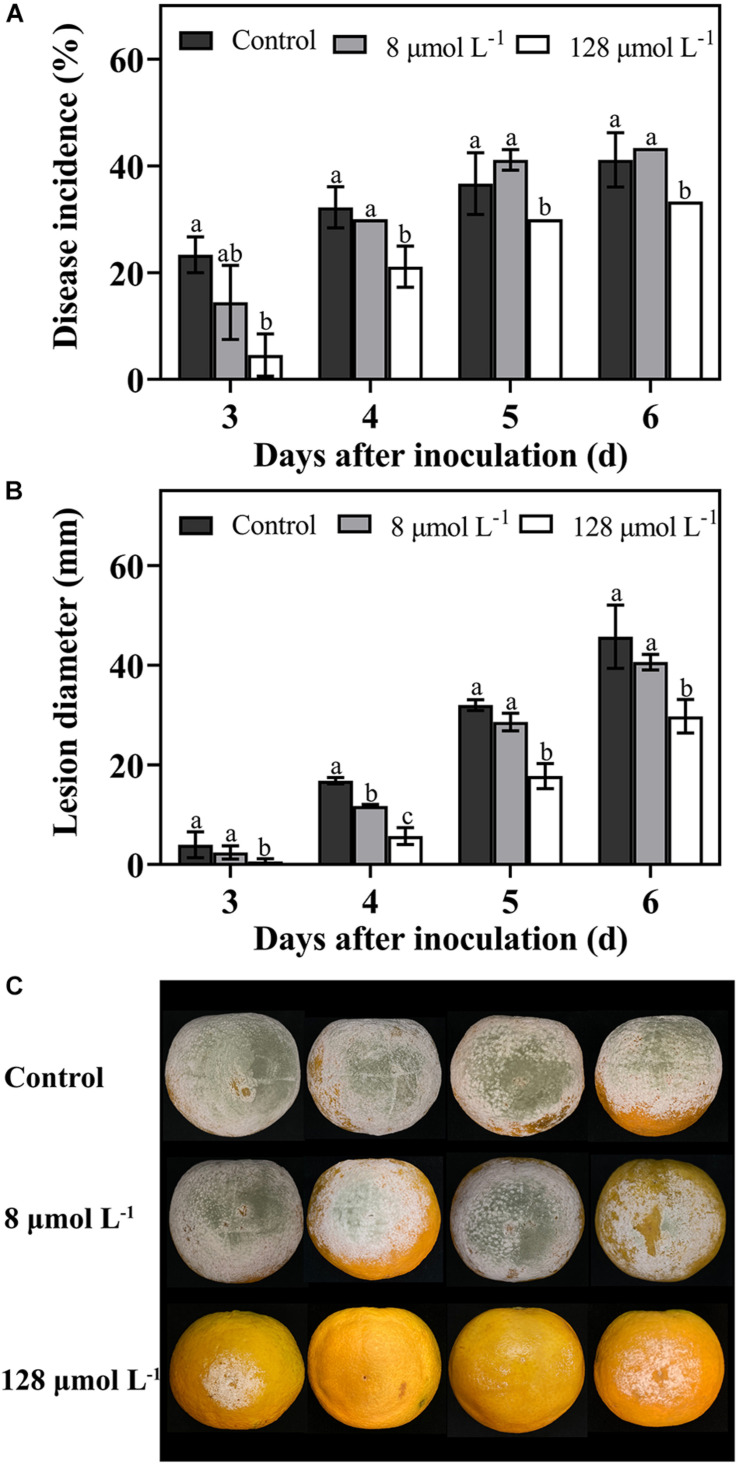
Effects of peptide thanatin on diseases incidence **(A)** and lesion diameter **(B)** of citrus fruit infected by *P. digitatum*. The images were the citrus fruit incubated at 25°C for 6 days **(C)**. The *P. digitatum* spore (5 × 10^4^ CFU mL^– 1^) were treated with thanatin with 0 (sterile water), 8 μmol L^– 1^ and 128 μmol L^– 1^ for 2 h at room temperature. Bar represented the SD of three independent experiments. The letters ‘a, b, and c’ indicated significant differences at the 0.05 level.

The *in vivo* test was carried out to determine the efficiency of thanatin against green mold caused by *P. digitatum* in citrus fruit. In this work, during the storage from 3 days to 6 days, the diseases incidence (Figure 2A), and lesion diameter (Figure 2B) of citrus fruit were significantly decreased after thanatin treatment with 128 μmol L^–1^, whereas 8 μmol L^–1^ did not exhibit obvious inhibition.

### Transcriptome Sequencing Quality

The RNA-seq primary quality results of control and treatment groups were summarized in Table 1, and the complete data were listed in Supplementary Table 2. In this work, a mean of 64.8 million and 59.2 million raw reads were obtained from control and treatment samples, respectively. After a strict quality control test, we received an average of 9.44 G (control) and 8.63 G clean data (thanatin). These reads were aligned on the reference genomes (*Penicillium digitatum* Pd1), and the total mapping ratio of each sample was about 94%. The uniquely and exon regions mapping portion of each sample were more than 93% and 75%, corresponding to the standard for transcriptional analysis.

**TABLE 1:** The summary reads of transcriptomic of *P. digitatum* libraries^1^.

**Parameter**	**C-1**	**C-2**	**C-3**	**T-1**	**T-2**	**T-3**
Raw-reads	64,407,606	65,682,414	64,292,320	55,484,254	55,111,192	67,139,776
Clean-reads	63,384,360	64,265,484	61,162,978	52,390,752	54,195,700	65,998,650
Clean-bases	9.51 G	9.64 G	9.17 G	7.86 G	8.13 G	9.90 G
Total mapped	60,122,323 (94.85%)	61,136,459 (95.13%)	57,402,722 (93.85%)	49,413,280 (94.32%)	51,200,443 (94.47%)	62,430,081 (94.59%)
Uniquely mapped	59,759,349 (94.28%)	60,765,054 (94.55%)	56,982,405 (93.16%)	49,078,110 (93.68%)	50,834,965 (93.8%)	61,948,522 (93.86%)
Exon mapped	6,839,552,794 (75.9955%)	7,018,423,052 (76.6992%)	6,475,463,773 (75.4011%)	5,600,277,800 (75.7663%)	5,898,175,695 (76.96%)	7,116,425,073 (76.1562%)

*^1^The samples C-1, C-2, C-3 were three replicates in control groups, and the three samples (T-1, T-2, and T-3) belonged to the groups treated with peptide thanatin at 4 μmol L^–1^ for 2 h.*

### Analysis of Overall Differential Expression Genes

**FIGURE 3:**
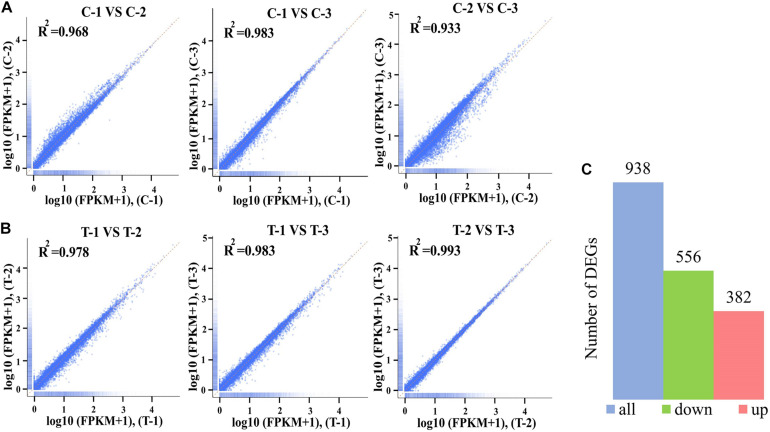
The square of the Pearson correlation coefficient (*R*^2^) among three biological replicates of control groups **(A)** and treatment groups at 4 mol L^– 1^ **(B)**. The Number of differentially expressed genes (DEGs) between control and treatment groups **(C)**. The *x*-axis and *y*-axis indicated the normalized Per Million mapped reads (FPKM) values from the control groups including C-1, C-2, and C-3 **(A)** and the treatment groups T-1, T-2, and T-3 **(B)**.

The gene expression patterns between treatment and control groups are presented in Figure 3. The square of the Pearson correlation coefficient (*R*^2^) corresponding to each biological replicates of control were over 0.93 (Figure 3A), and the number corresponding to treated samples was over 0.97 (Figure 3B), such high *R*^2^ indicated high credibility of biological repetition in the present work. A total of 9408 genes were transcribed between control and treatment groups. Based on a threshold, there were 938 DEGs between treated panel and control group, including 556 upregulated, and 382 downregulated DEGs, respectively, (Figure 3C).

### Enrichment Analysis of Gene Ontology

**FIGURE 4:**
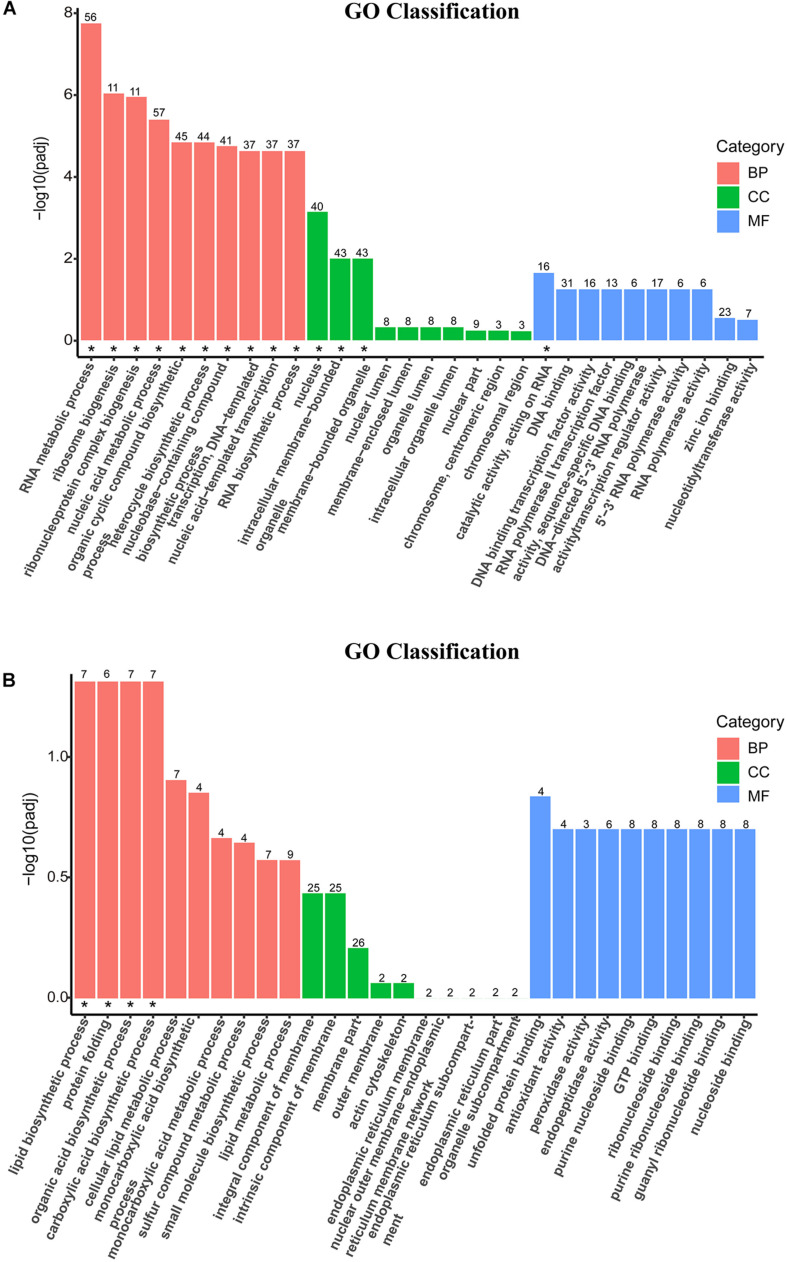
Secondary entry bar chart of down-GO terms **(A)** and up-GO terms **(B)** mapped with Gene Ontology (GO) functional classification. The terms on the *x*-axis were the top 10 GO categories of biological process (BP), cellular component (CC), and molecular function (MF), respectively. The *y*-axis was the degree of statistical difference, and the symbol “*” located at the figure indicated the enriched GO term was at padj < 0.05.

The DEGs were mapped into the GO database and divided into three categories: biological process (BP), cellular component (CC), and molecular function (MF). The top 10 GO terms in the three categories of downregulated DEGs (Figure 4A) and upregulated DEGs (Figure 4B) were statistically different (padj < 0.05). The enriched GO categories (padj < 0.05) are listed at the Supplementary Table 3. According to the BP categories, the down-categories were mainly classified into RNA metabolism related processes, including RNA metabolic process; ribosome biogenesis; ribonucleoprotein complex biogenesis; nucleic acid metabolic process; nucleobase-containing compound biosynthetic process; transcription, DNA-templated; nucleic acid-templated transcription and RNA biosynthetic process. In addition, the thanatin stress on RNA significantly affected nucleus and catalytic activities. By analyzing the subordination of a series categories on the Ontobee web^3^ (Ong et al., 2017), the transcription, DNA-templated; ncRNA processing; rRNA processing; nucleus; catalytic activity, acting on RNA were the most specific GO categories associated with the RNA metabolism (Supplementary Table 3). A total of four up-categories (BP) associated with metabolic processes were enriched, including: lipid biosynthetic process, organic acid biosynthetic process, carboxylic acid biosynthetic process and protein folding.

### Kyoto Encyclopedia of Genes and Genomes Pathway Enrichment Analysis of DEGs

**FIGURE 5:**
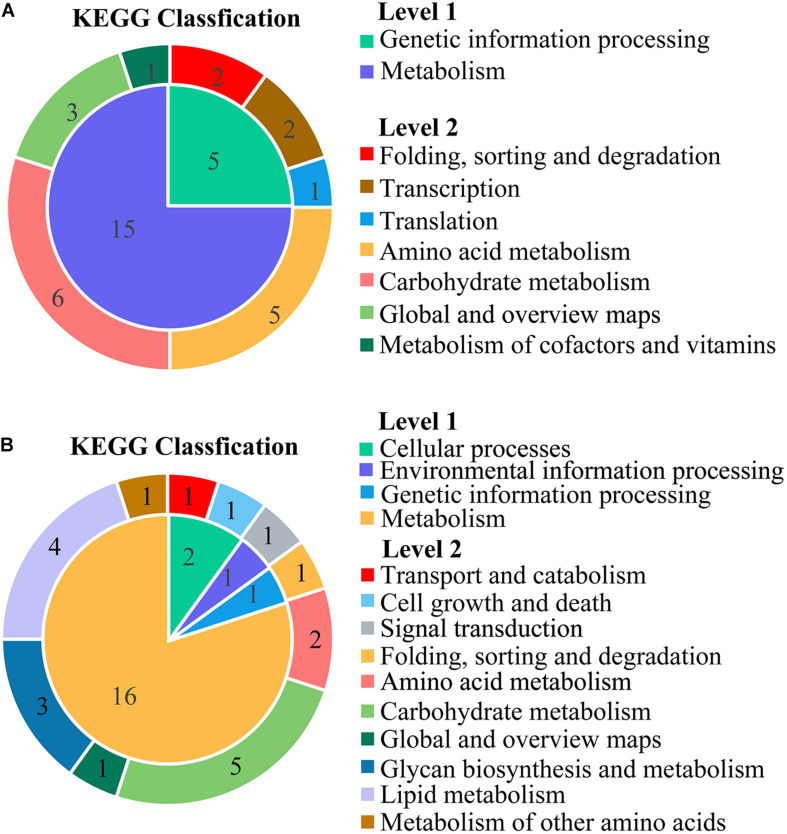
The classification for the top 20 enriched Kyoto Encyclopedia of Genes and Genomes (KEGG) pathways mapped with the downregulated DEGs **(A)** and upregulated DEGs **(B)**. The inner ring was the systems (level 1), and the outer ring was the subcategories (level 2). All the names (level 1, level 2) were described at the vertical row. The numbers in the region of ring represented the number of KEGG pathways (level 3).

Kyoto Encyclopedia of Genes and Genomes pathway analysis was performed to uncover enriched biochemical pathways mapped by DEGs. The complete data of the top 20 pathways were shown in the Supplementary Table 4. The various subcategories (level 2) were grouped by the top 20 enriched pathways (level 3) and clustered into several systems (level 1) in KEGG (Figure 5).

The KEGG subcategories of genetic information processing were annotated to downregulated DEGs, including the following: folding, sorting and degradation (sulfur relay system; protein export), transcription (RNA polymerase; basal transcription factors), and translation (ribosome biogenesis in eukaryotes). For the metabolism, four subcategories were clustered by 15 down-pathways and grouped into amino acid metabolism (glycine, serine, and threonine metabolism; valine, leucine, and isoleucine degradation; alanine, aspartate, and glutamate metabolism; cysteine and methionine metabolism; valine, leucine, and isoleucine biosynthesis). Additionally, the subcategories of carbohydrate metabolism were inhibited, including glyoxylate and dicarboxylate metabolism, propanoate metabolism, pentose and glucuronate interconversions, pentose phosphate pathway, fructose and mannose metabolism, and butanoate metabolism.

The up-pathways of the overexpressed DEGs were mainly assigned to a wide variety of metabolic processes in the sub-classification of lipid metabolism (glycerophospholipid metabolism, steroid biosynthesis, biosynthesis of unsaturated fatty acids, and sphingolipid metabolism). Some DEGs were upregulated in carbohydrate metabolism, such as amino sugar and nucleotide sugar metabolism, fructose and mannose metabolism, starch and sucrose metabolism, galactose metabolism, and glycolysis/gluconeogenesis.

**FIGURE 6:**
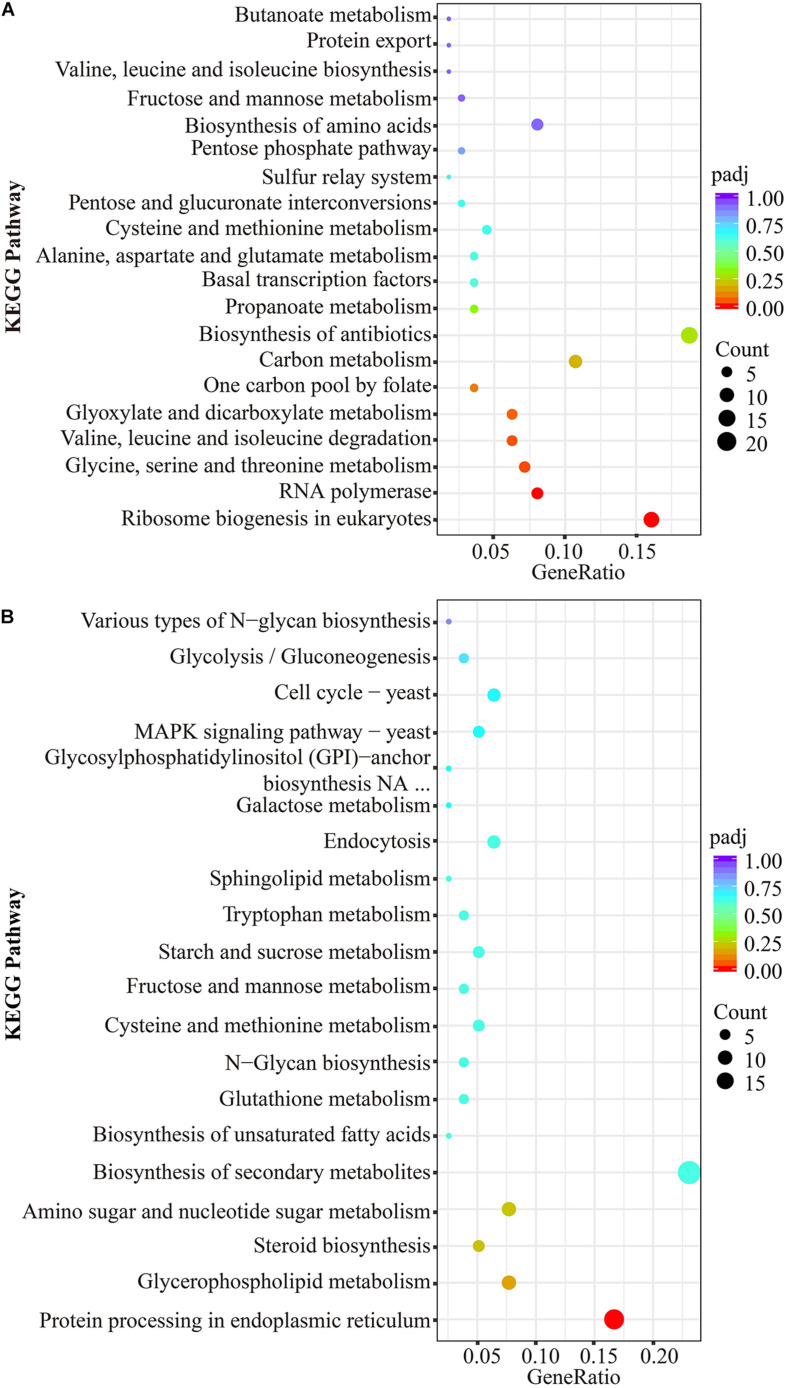
KEGG significant enrichment analysis for the top 20 pathways mapped with downregulated DEGs **(A)** and upregulated DEGs **(B)**. The ordinate represented KEGG pathway (level 3), the abscissa meant rich factor of genes, the size of dot was the count of genes mapped to corresponding pathways, and the less padj corresponding to red color indicated greater enrichment.

The scatter plots showed the enrichment degree of the top 20 down- (Figure 6A) and up-pathways (Figure 6B). It was found that DEGs participated in genetic information processing, and they were significantly enriched in Ribosome biogenesis in eukaryotes (26230971, 26230402, 26236159, 26235119, 26228459, 26228529, 26229743, 26235457, 26233954, 26235941, 26230478, 26229562, 26229368, 26231610, 26229081, 26236221, 26233986, and 26232110) and RNA polymerase (26230310, 26236983, 26236361, 26233723, 26233907, 26234379, 26230128, 26230714, and 26233384), which belongs to the subcategories of translation and transcription, respectively, (padj < 0.05). Many DEGs encoding amino acids biosynthesis (26230688, 26229585, 26234424, 26229949, 26233005, 26231449, 26232854, 26230010, 26229771, 26234736, 26235200, and 26234679) and amino acid metabolisms (26236295, 26236923, 26233364, 26233458, 26233454, 26234736, 26229949, 26231951, 26234833, 26237113, 26233928, 26232914, 26234690, 26235200, 26231423, 26232699, 26236483, 26235087, 26234424, 26233005, 26235997, 26229771, 26229585, 26228955, 26231449, 26230731, 26229040, 26231939, and 26233373) were affected by thanatin. Two amino acid metabolism pathways (Glycine, serine, and threonine metabolism; Valine, leucine, and isoleucine degradation) were seriously inhibited by thanatin (padj < 0.05). For the up-pathways, protein processing in endoplasmic reticulum was the only enriched pathway mapped with the DEGs (26230932, 26233811, 26233535, 26230135, 26230144, 26234598, 26233512, 26237258, 26229590, 26232080, 26230326, 26230462, 26232684, and 26232910).

### Verification of Differently Expressed Genes by Quantitative RT PCR

**FIGURE 7:**
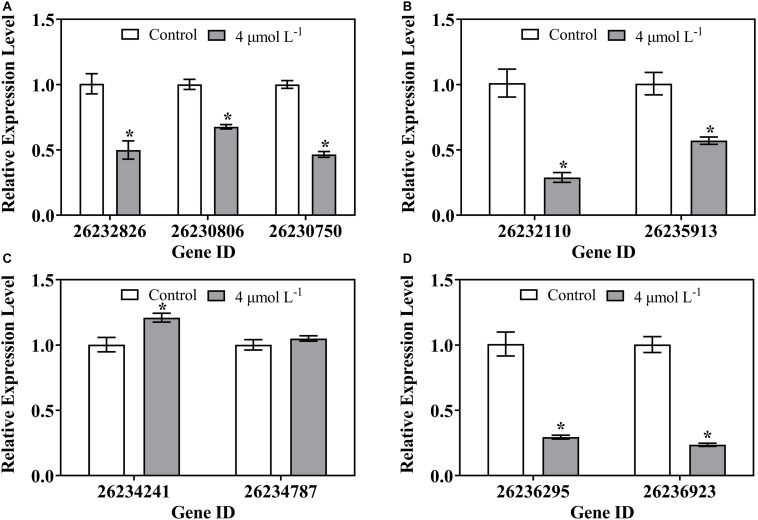
Gene expression of *P. digitatum* when exposed to thanatin related to Transcription **(A)**, Translation **(B)**, Folding, sorting and degradation **(C)**, and Amino acid metabolism **(D)**. The data were shown as the mean ± SD (*n* = 3), the “*” located on the columns (4 μmol L^–1^) represented statistical difference (*p* < 0.05) compared with control.

Nine DEGs mapped to the primary pathways were analyzed by qRT-PCR to validate the expression changes of genes. The expression of genes involved in the transcription (Figure 7A), translation (Figure 7B), and amino acid metabolism (Figure 7D) in treatment groups were significantly decreased compared to that in the control group. However, the transcriptional levels of gene encoding mRNA degradation (26234241) were remarkably increased with thanatin treatment (Figure 7C). The expression pattern of these genes was similar to transcriptome profile (Table 2).

**TABLE 2:** RNA-seq analysis of nine genes mapped to the most enrichment pathways.

**Gene ID**	**Gene name**	**Description**	**Fold change (test/control)**
**Transcription**			
26232826	PDIP_45080	Transcription initiation factor TFIIE, beta subunit, putative	0.63 (down)
26230806	PDIP_24840	SART1, putative	0.77 (down)
26230750	PDIP_24280	RNA polymerase II Elongator complex associated protein Kti12, putative	0.65 (down)
**Translation**			
26232110	PDIP_37920	Non-sense-mediated mRNA decay protein 3	0.45 (down)
26235913	PDIP_75970	Eukaryotic translation initiation factor 3 subunit EifCk, putative	0.73 (down)
**Folding, sorting and degradation**			
26234241	PDIP_59250	Decapping enzyme Dcp2, putative	2.03 (up)
26234787	PDIP_64710	Mitochondrial Hsp70 chaperone (Ssc70), putative	1.42 (up)
**Amino acid metabolism**			
26236295	PDIP_79790	3-methyl-2-oxobutanoate dehydrogenase, putative	0.33 (down)
26236923	PDIP_86090	Biotin-dependent 2-oxo acid dehydrogenases acyltransferase, putative	0.37 (down)

### Effect of Thanatin on *P. digitatum* DNA and RNA Synthesis

**FIGURE 8:**
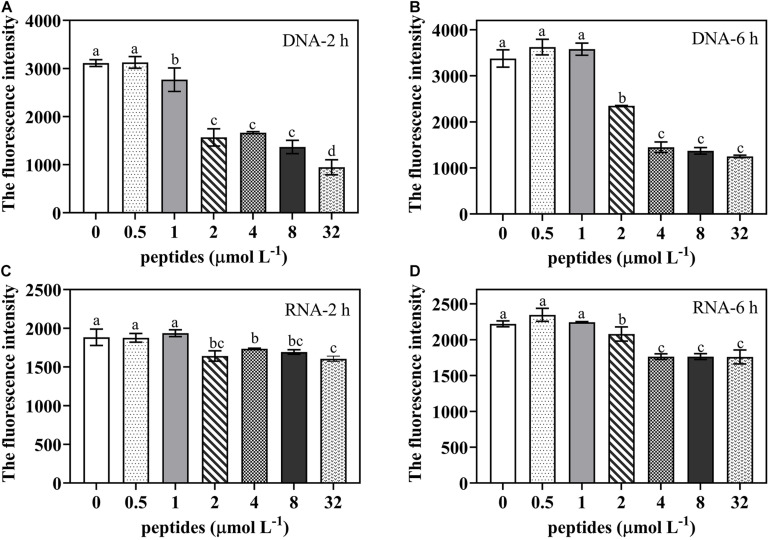
The DNA/RNA content of *P. digitatum* spore (5 × 10^4^ CFU mL^–1^) treated without or with thanatin at various concentration for 2 h **(A,C)** and 6 h **(B,D)**. The data were shown as the mean ± SD (*n* = 3), the different letters “a,” “b,” “c,” and “d” indicated significant differences at *p* < 0.05.

To identify potential effect of thanatin on nucleic acid synthesis of *P. digitatum*, the DAPI dye was used to quantify DNA and RNA content (Figure 8). The results revealed that, compared to the control group, the DNA and RNA contents were obviously reduced when treated with thanatin at various concentration from 2 up to 32 μmol L^–1^ for 2 and 6 h.

### Effect of Thanatin on *P. digitatum* Protein Synthesis

To further investigate whether protein synthesis was impaired by the thanatin, we determined the soluble protein content of *P. digitatum*. The soluble protein content of *P. digitatum* was significantly decreased with the thanatin concentration above 2 μmol L^–1^ for 2 h and 6 h. The average soluble protein content at 2, 4, and 8 μmol L^–1^ were 4.40, 3.26, and 2.57 μg, respectively, which were much lower than that in control samples (6.03 μg) after the incubation of 2 h.

## Discussion

*Penicillium digitatum* is one of the most destructive pathogens in citrus fruit. Like other peptides, the insect peptide thanatin exerted prominent inhibition and lethal effect on *P. digitatum* spores *in vitro* test (Munoz et al., 2007; Luz et al., 2017; Li et al., 2019). The results showed that better antifungal effect required higher dosage and longer incubation time (Figure 1). And the MFC (128 μmol L^–1^) was effective to decrease the fruit decay caused by green mold (Figure 2). In addition, thanatin could also reduce sour rot caused by *G. citri-aurantii*, and show lower hemolysis than commercial fungicides (Liu et al., 2019). These finding indicated thanatin could be a potential promising candidate to substitute conventional chemical agents to control citrus green mold and sour rot disease during postharvest.

To investigate the molecular influence of *P. digitatum* when exposed to thanatin, the GO term enrichment and KEGG pathway of transcriptomic profiles were deeply analyzed.

Transcription is the first step of gene expression procedure that synthesize RNAs from DNA templates. The RNA polymerases involved in RNA synthesis have been reported to be targets of AMPs such as microcin J25 and capistruin (Braffman et al., 2019). RNA polymerase I acts on transcription of rRNA, while 5S rRNA, tRNA, and U6 spliceosomal snoRNA are transcribed by RNA polymerase III, and RNA polymerase II is responsible for mRNA and non-coding RNA (Richard and Manley, 2009). In our study, the genes encoding for RNA polymerase I, RNA polymerase III, and relative RNA complex were downregulated by thanatin. In eukaryotic transcription, the basal transcription factors (BTFs) play an important role in activating the function of RNA polymerases (I -III) at transcription sites. Under thanatin stress, the expression level of DEGs related to BTFs were downregulated 1.35- to 2.04-fold, including *TBP* (26231193), *TFIIE2* (26232826), *TFIIH1* (26232621), and *TAF3* (26228750). Among them, TFIIE2 is the small beta submit of TFIIE binding to the core region of promoter upstream of the transcription initiation site, and it can be firmly combined with RNA polymerase II or other BTFs (Blanco and Blanco, 2017). *TFIIE2* (26232826) was significantly suppressed by thanatin based on the qRT-PCR results (Figure 7A). In addition, other genes (*RRN3, Med8, Kti12*) encoding some complex functions of RNA polymerase (I, II) were downregulated as well. Kti12 is a protein related to RNA polymerase II elongator complex, it is responsible for the transcription elongation and tRNA modification activity (Okuda et al., 2000). Both RNA-seq and qRT-PCR results showed *Kti12* (26230750) was obviously downregulated, implying the disfunction of RNA polymerase II occurred by thanatin (Figure 7A). The results therefore suggested that thanatin suppressed transcription processes by downregulating the genes associated with RNA polymerases and BTFs.

Translation is responsible for the synthesis of protein from RNA. Ribosome is a complex and large molecular machine that can work with mRNA and tRNA to construct proteins, and it is composed of many large subunits and small submits, including four rRNAs and ∼80 ribosomal proteins (Krutyhołowa et al., 2019). Through KEGG pathway analysis, a total of 18 downregulated genes were enriched in the pathway of ribosome biogenesis in eukaryotes. The ribosome biogenesis is the most complex and energy-consuming cellular process that requires abundant ribosomal proteins, rRNA, rRNAs assembly factors, and so on. In addition, ribosome biogenesis is a co-transcription process with rRNA synthesis, and rRNA plays a critical structural and functional role in constituting the structure of ribosomes (de la Cruz et al., 2015; Cheng et al., 2017). The 90S particle is the largest type of early ribosomal particles, which consists of various pre-rRNAs, assembly factors, snoRNAs, and a series of ribosomal proteins (Kornprobst et al., 2016). A total of twelve genes (*UTP22, Rrp7, UTP5, UTP15, UTP4, NAN1, UTP13, Dip2, UTP6, Imp4, Bms1*, and *Rc11*) related to the 90S pre-ribosome components were downregulated 1.87- to 5.30-fold in thanatin-treated *P. digitatum*. We analyzed these genes more deeply and found that all the genes encoded U3 small nucleolar RNA-associated proteins (U3 snoRNPs). U3 snoRNP is the core component of 90S particle and can induce the first step to stabilize the ribosome primary structure and then recruit proteins for the structure of 90S particle (Chaker-Margot et al., 2017). Furthermore, U3 snoRNP is a chaperone for pre-18S rRNA folding while pre-18S rRNA can maintain the structure of pre-40S and control the site cleavage of rRNA processing (Dutca et al., 2011). These downregulated genes indicated that the construction of ribosome core structure was blocked by thanatin.

Additionally, the other downregulated genes (*NOP4, Nug1/2, NMD3*, and *RIO1*) involved in the ribosomal biogenesis are responsible for encoding pre-60S particles or pre-40S particles. Protein kinase RIO1 is essential for the final maturation step of ribosomal small subunit converting from the pre-rRNA into the mature 18S rRNA (LaRonde, 2014). The *RIO1* (26236159) was found to be 2.98-fold reduced by thanatin. Only three genes (26235423, 26235982, and 26232506) encoding for the mature structure of ribosome large subunit were found to be affected by thanatin, implying that thanatin mainly targeted on the construction of primary structure of ribosome, rather than the assembly process of mature subunits. As the translation initiation is the rate-limiting factor of the entire translation process in eukaryotes (Arava et al., 2003). Previous studies have found that AMPs Bac71-35, oncocins, and apidaecins disrupted the procedure by blocking the peptide exit tunnel (Krizsan et al., 2014; Gagnon et al., 2016). In this work, except for *HexA* (26233464), the DEGs related to translation initiation factor (26228980, 26231287, 26228327, and 26235913) and aminoacyl-tRNA biosynthesis (26230368 and 26232778) were downregulated. In addition, more than 20 downregulated genes participated in various pathways with amino acid biosynthesis and metabolism, such as valine, leucine, and isoleucine degradation; glycine, serine, and threonine metabolism; and alanine, aspartate, and glutamate metabolism. The results indicated the possible mechanisms of thanatin to disturb the normal translation procedure of *P. digitatum*.

Fungi could enhance the xenobiotic detoxification ability and decrease the intracellular drug level by activating the drug efflux transporters under abiotic stress to develop resistance to multiple drugs (Morschhäuser, 2010). The transporters of major facilitator superfamily (MFS) and ATP-binding cassette (ABC) of microorganisms that can efflux the xenobiotics were known as the fungi drug-resistance basis (Paul and Moye-Rowley, 2014). These transporters facilitate the macromolecules, ions, or small molecules across a biological membrane to serve for the own physiological state to grow. On the other hand, they can interact with the drugs as a target that may contribute to the development of drug resistance (Perlin et al., 2014). Previously, it was reported that a serious of genes encoding MFS (Wang et al., 2019) or ABC (Yang et al., 2016) were affected in response to antifungal agents, which implied a potential risk of resistance to the used antimicrobials. We analyzed the changes of DEGs encoding MFS were affected by thanatin. The MFS is a class of transport proteins that plays an important role in many substrate transports in cells, and it can pump off the fungal toxins, thereby enhance the ability of *P. digitatum* to infect the plant host. In addition, the overexpression of genes encoding MFS transporter conferred the drug-resistance of fungi (Wu et al., 2016; de Ramón-Carbonell et al., 2019). In this work, the genes encoding MFS monosaccharide (26236839), sugar (26228725), peptide (26233853) transporter were downregulated by 2.03, 1.48, and 1.82-fold, respectively. And the other genes encoding MFS (26229980, 26235045, 26228932, 26236120, 26232765, 26236803, 26232545, and 26229143) were downregulated under thanatin stress from 1.39- to 5.99-fold, implying thanatin could potentially impair the detoxification and drug resistance development in *P. digitatum*. ABC transporter genes in *P. digitatum* are directly involved in drug resistance. (Sanchez-Torres and Tuset, 2011). The genes encoding ABC transporter (26237078, 26230837, 26230246, 26234595, and 26233626) were affected under thanatin treatment, which were unfavorable to alleviate the environment stress in *P. digitatum*. Besides, arb1 (26233626), an ABC transporter ATP-binding protein that functions as an ATPase involving in the 40S and 60S ribosomal biogenesis (Dong et al., 2005), was downregulated by 2.36-fold, which is also corresponding to the block of ribosomal biogenesis induced by thanatin. The antifungal peptides were recognized as promising candidates for traditional chemical fungicides because of their multiple targets of fungal, reducing the propensity of resistance development (Bradshaw, 2003; Fernández de Ullivarri et al., 2020). In this research, although various genes encoding for MFS and ABC transporter were affected by thanatin, which implied the development block of drug resistance, the further experiments are still required to confirm.

In addition, other transcriptomic alterations of *P. digitatum* related to stress response were found in treatment group. Heat shock proteins (HSPs) are important in stabilizing proteins, and they are recognized as molecular chaperones in response to stressful environmental conditions (Tiwari et al., 2015). In this study, the DEGs (26230932, 26233811, 26230144, 26235140, 26231366, 26230266, 26228880, 26233906, 26231397, 26236477, and 26231735), which belongs to HSPs, such as *hsp70*, *hsp90*, *hsp78*, and *hsp60*, were upregulated from 1.26- to 2.47-fold. Under exogenous treatment, the level of reactive oxygen species (ROS) in the cells may rapidly increase, and the excessively generated toxic oxygen radicals can react with the essential intracellular biomolecules such as nucleic acids, proteins, lipid causing oxidative injury, and further leading to cell damage and death. Moreover, ROS also act as a signaling molecule in cascades (Sauer et al., 2001). It has been reported that the peptide cathelicidin could effectively inhibit the growth of bacteria mainly due to the induced accumulation of ROS in cells (Rowe-Magnus et al., 2019). In this work, an increase of expression level of DEGs related to glutathione (26230731, 26235672, 26234900, and 26229514), thioredoxin (26234374, 26236481, and 26230522) and peroxisome (26233734 and 26234690) was observed, implying thanatin can interfere with ROS homeostasis by activating key antioxidative systems but need further exploration.

**FIGURE 9:**
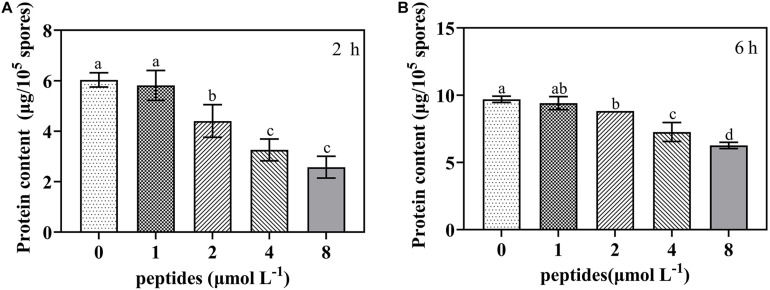
The soluble protein content of *P. digitatum* spore (1 × 10^5^ CFU mL^–1^) treated without or with thanatin at various concentration for 2 h **(A)** and 6 h **(B)**. The data were shown as the mean ± SD (*n* = 3), the different letters “a,” “b,” “c,” and “d” indicated significant differences at *p* < 0.05.

Based on the RNA-seq results, the potential action of the thanatin on genetic transmission process was further verified. Previous research suggested that, the process of spore germination and mycelium formation under suitable nutritional conditions are usually accompanied by multiple metabolic activities such as respiration, RNA, and protein synthesis (d’Enfert, 1997; Osherov and May, 2001; van Leeuwen et al., 2013; Zhou et al., 2018a). In present study, DNA and RNA contents of *P. digitatum* were decreased by thanatin at above 2 μmol L^–1^ (Figure 8), implying that thanatin could inhibit DNA and RNA synthesis of *P. digitatum*. Since the binding strengths of DAPI to RNA and DNA are different, the strength of DAPI to RNA is 20% of that to DNA, it was difficult to compare the inhibitory effect of thanatin on the synthesis of DNA and RNA in this work. Proteins are the primary participants within the cell that conducted various functions specified by the encoding genes. Studies have found that protein synthesis is essential for spore germination and mycelia basic cellular machinery (Riquelme et al., 2018). The soluble protein content of *P. digitatum* were decreased by thanatin treatment at above 2 μmol L^–1^ (Figure 9). Combined with transcriptional analysis, the reason for the decline in protein production may be due to lack of amino acids and inhibition of ribosomal biogenesis in *P. digitatum* under thanatin. Overall, these results were consistent with the RNA-seq analysis results that shown downregulation of the expression of the genes involved in genetic transmission process, especially in transcription and translation.

## Conclusion

In summary, thanatin was effective to inhibit the growth of *P. digitatum in vitro* and *in vivo*, and the antifungal action mode against *P. digitatum* at molecular level were unraveled. Specially, the genes encoding RNA polymerases, ribosome biogenesis, and amino acid metabolisms were downregulated, and the DNA, RNA, and protein content were reduced significantly, suggesting that thanatin has an effect on information transmission of *P. digitatum*. Furthermore, the present work demonstrates transcriptome provide a powerful and feasible tool for understanding the various action mechanisms of peptides.

## Data Availability Statement

All the raw data were deposited in NCBI’s Sequence Read Archive. The six sequences were grouped to a BioProject (PRJNA646579): Penicillium digitatum raw sequence reads, and the six SRA accession numbers were listed as follows: SRR12245251, SRR12245252, SRR12245250, SRR12245253, SRR12245249, and SRR12245254 (https://www.ncbi.nlm.nih.gov/bioproject/PRJNA646579/).

## Author Contributions

KZ: conceptualization. GF and XL: methodology. WW: software. GF and WW: validation. GF: formal analysis. GF and XL: investigation. KZ and LD: resources. GF: data curation. GF and WW: writing (original draft preparation). GF and WW: writing (review and editing). XL: visualization. KZ: supervision. KZ and LD: project administration. KZ and LD: funding acquisition. All authors have read and approved this version of the article.

## Conflict of Interest

The authors declare that the research was conducted in the absence of any commercial or financial relationships that could be construed as a potential conflict of interest.

## Footnotes

1 http://fungi.ense-mbl.org/Penicillium_digitatum_pd1_gca_000315645/Info/Index

2 http://www.ncbi.nlm.nih.gov/tools/primer-blast/

3 http://www.ontobee.org/

## Supplementary Material

Supplementary Table 1 The primer sequences of *P. digitatum* genes when exposed to thanatin at 0 or 4 μmol L^–1^ for the validation by qRT-PCR.

Supplementary Table 2 Sequencing quality control of the data, and the mapping rate to the reference genomes.

Supplementary Table 3 Total significantly enriched GO terms (padj < 0.05).

Supplementary Table 4 The RNA-seq data of the top 20 downregulated and upregulated pathways. The “^∗^” symbol located behind the pathways (level 3) meant the pathway was enriched significantly (padj < 0.05, padj value was the multiple hypothesis-corrected *p* value).
